# Snare-Assisted Management of Nose Cone Entrapment During Self-Expandable Transcatheter Aortic Valve Replacement

**DOI:** 10.1016/j.jaccas.2024.103120

**Published:** 2025-02-05

**Authors:** Vu Hoang Vu, Thanh Cong Nguyen, Dinh Hoang Nguyen, Thang Duc Ho, Binh Quang Truong

**Affiliations:** a Faculty of Medicine, University of Medicine and Pharmacy at Ho Chi Minh City, Ho Chi Minh City, Vietnam; b Department of Interventional Cardiology, University Medical Center Ho Chi Minh City, Ho Chi Minh City, Vietnam; c Department of Adult Cardiovascular Surgery, University Medical Center, Ho Chi Minh City, Vietnam

**Keywords:** aortic valve stenosis, nose cone entrapment, snare technique, TAVR

## Abstract

Nose cone entrapment is a rare but serious complication of transcatheter aortic valve replacement (TAVR) that requires prompt recognition and management to prevent adverse outcomes. A 77-year-old man with severe aortic stenosis underwent TAVR with a 29-mm Evolut R valve (Medtronic). The nose cone became lodged at the distal stent frame as a result of infolding, caused by a calcified nodule. Standard maneuvers failed, but the complication was successfully resolved using a snare device deployed through contralateral femoral artery access without damage to the prosthesis. Few similar cases have been reported. This case underscores the importance of recognizing risk factors such as severe calcification, significant annular angulation, and large-diameter valves. It also highlights the snare technique as an appropriate, minimally invasive solution for resolving nose cone entrapment. The snare technique is a reliable approach to manage nose cone entrapment during TAVR.

## Graphical Abstract

**Figure:**
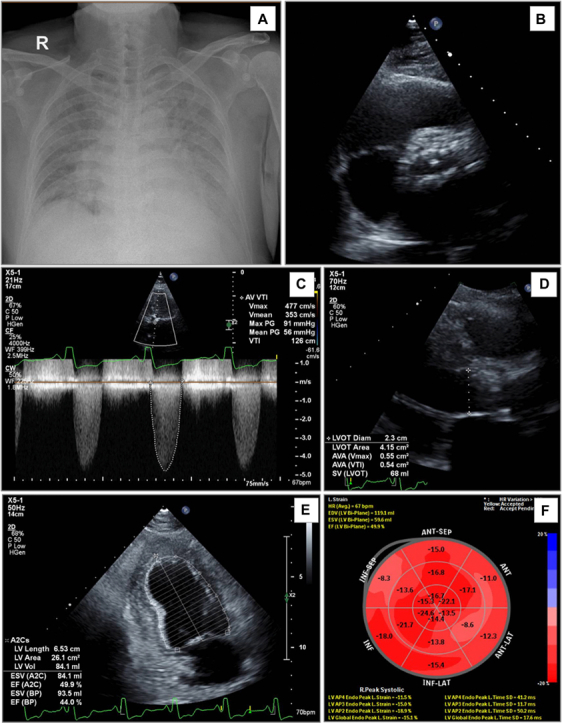

## History of Presentation

A 77-year-old man presented with a 1-week history of chest pain and dyspnea on exertion. Three days before admission, he experienced worsening substernal chest pain at rest, associated with sweating and palpitations, despite outpatient treatment. On presentation, he showed signs of acute heart failure, including orthopnea and an oxygen saturation of 90% on room air. His body mass index was 27 kg/m^2^, and no peripheral edema was present. Cardiac examination revealed a systolic murmur at the left sternal border radiating to the neck, along with bilateral moist rales at the lung bases.

**Take-Home Messages**

• Early recognition of nose cone entrapment risk during TAVR is crucial for preventing life-threatening complications.
• In the event of entrapment, avoiding aggressive maneuvers and using support devices such as a snare can ensure swift and effective resolution while minimizing damage to the valve and surrounding structures.

## Past Medical History

The patient had a history of 2-vessel coronary artery disease, with stents placed in the proximal left anterior descending (LAD) coronary artery and right coronary artery (RCA) 2 years earlier. He also received a diagnosis of severe aortic valve stenosis but declined invasive treatment. Over the past 6 months, recurrent dizziness led to the diagnosis of bilateral carotid and vertebral artery stenosis, treated with stenting. Despite these interventions, the dizziness persisted, culminating in a fall 2 months earlier that resulted in a left-sided subdural hematoma and cerebral contusion.

## Differential Diagnosis

The combination of peripheral hypoperfusion, acute heart failure, and chest pain strongly pointed toward severe aortic stenosis as the primary diagnosis. Although atrial fibrillation could exacerbate these symptoms, acute coronary syndrome remained an important differential diagnosis that required exclusion.

## Investigations

**Figure 1:**
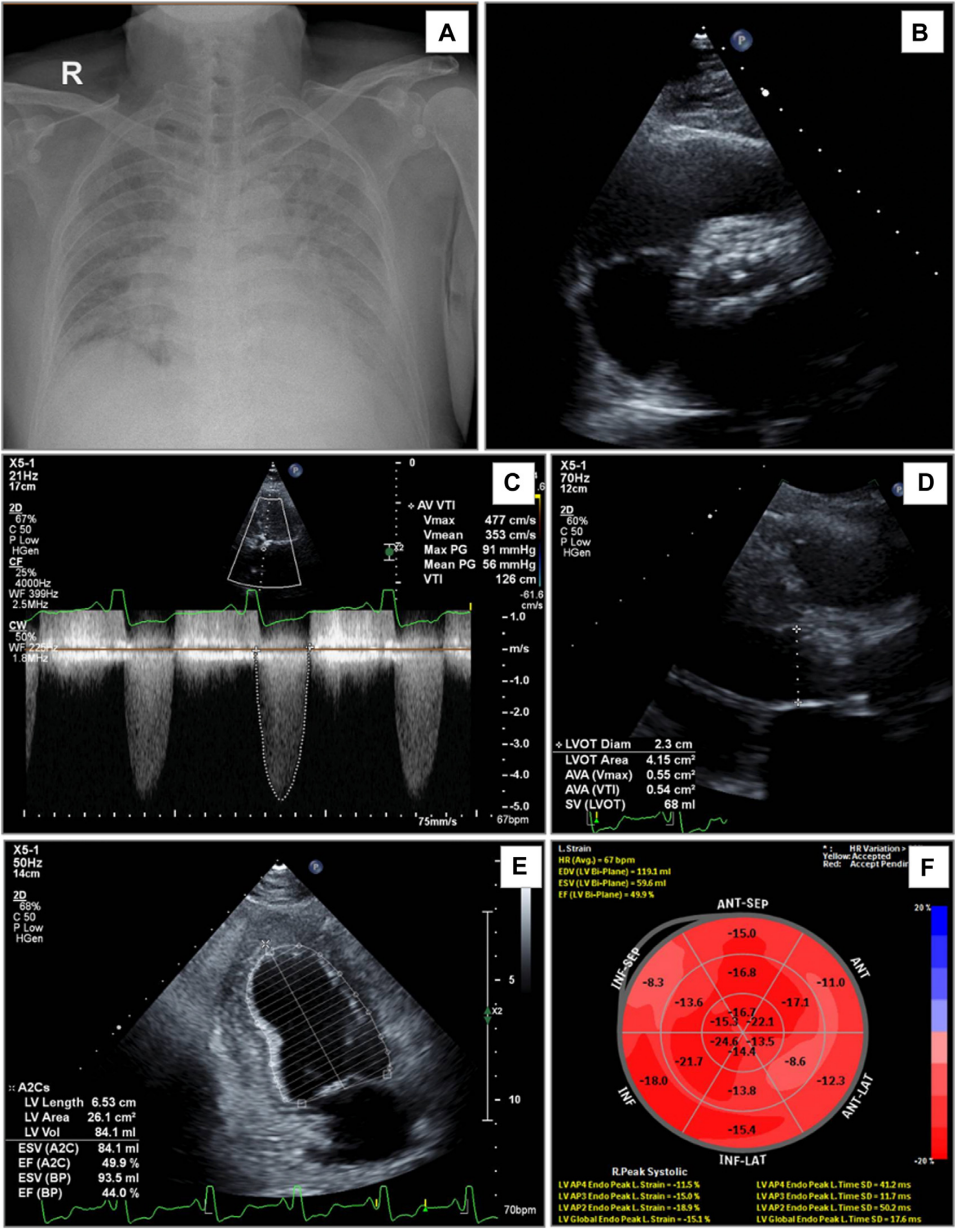
Chest Radiography and Transthoracic Echocardiography (A) Chest radiography demonstrates bilateral pulmonary congestion. (B to F) Transthoracic echocardiography reveals a severely calcified trileaflet aortic valve with significant stenosis (maximum velocity [Vmax], 4.77 m/s; aortic valve area [AVA], 0.54 cm^2^), mildly reduced left ventricular (LV) systolic function (ejection fraction, 44%), and mild left ventricular strain reduction. ANT = anterior; AV = aortic valve; C = compression; CF = color flow; CW = continuous wave; EDV = end-diastolic volume; EF = ejection fraction; ESV = end-systolic volume; H = harmonics; HR = heart rate; INF = inferior; LAT = lateral; LVOT = left ventricular outflow tract; P = persistence; PG = pressure gradient; SEP = septal; SV = stroke volume; Vmean = mean velocity; VTI = velocity time integral; 2D = 2-dimensional.

Laboratory test results showed a mildly elevated high-sensitivity troponin T level (30.1 ng/L) and a significantly increased N-terminal pro–B-type natriuretic peptide (NT-proBNP) concentration (6,412 ng/L). Serial electrocardiograms indicated paroxysmal atrial fibrillation with rapid ventricular response and left bundle branch block. A chest radiograph revealed bilateral pulmonary congestion (Figure 1A). During hospitalization, episodes of atrial fibrillation were associated with worsening dyspnea, chest pain, and hypotension.

Echocardiography confirmed severe aortic stenosis with a heavily calcified trileaflet valve, featuring an aortic valve area of 0.54 cm^2^, a maximum pressure gradient of 91 mm Hg, and a mean pressure gradient of 56 mm Hg. Moderate aortic regurgitation was present, with a vena contracta width of 4.5 mm. The left ventricle showed significant hypertrophy and dilation, indicated by an end-diastolic diameter of 59.4 mm and an end-diastolic volume of 167 mL. Systolic function was impaired, with an ejection fraction of 44%, and global longitudinal strain was mildly reduced (−15.1%). Diastolic function was classified as grade II, with an E/e’ ratio (the ratio of the peak early mitral inflow velocity [E] to the early diastolic mitral annular velocity [e']). of 13.2, along with mild tricuspid regurgitation and an elevated pulmonary artery pressure of 55 mm Hg (Figures 1B to 1F). Multislice computed tomography showed no restenosis in the LAD artery and RCA stents, and the aortic valve calcium score was 4,666. Collectively, these findings confirmed severe aortic stenosis as the underlying cause of the patient’s symptoms.

**Figure 2:**
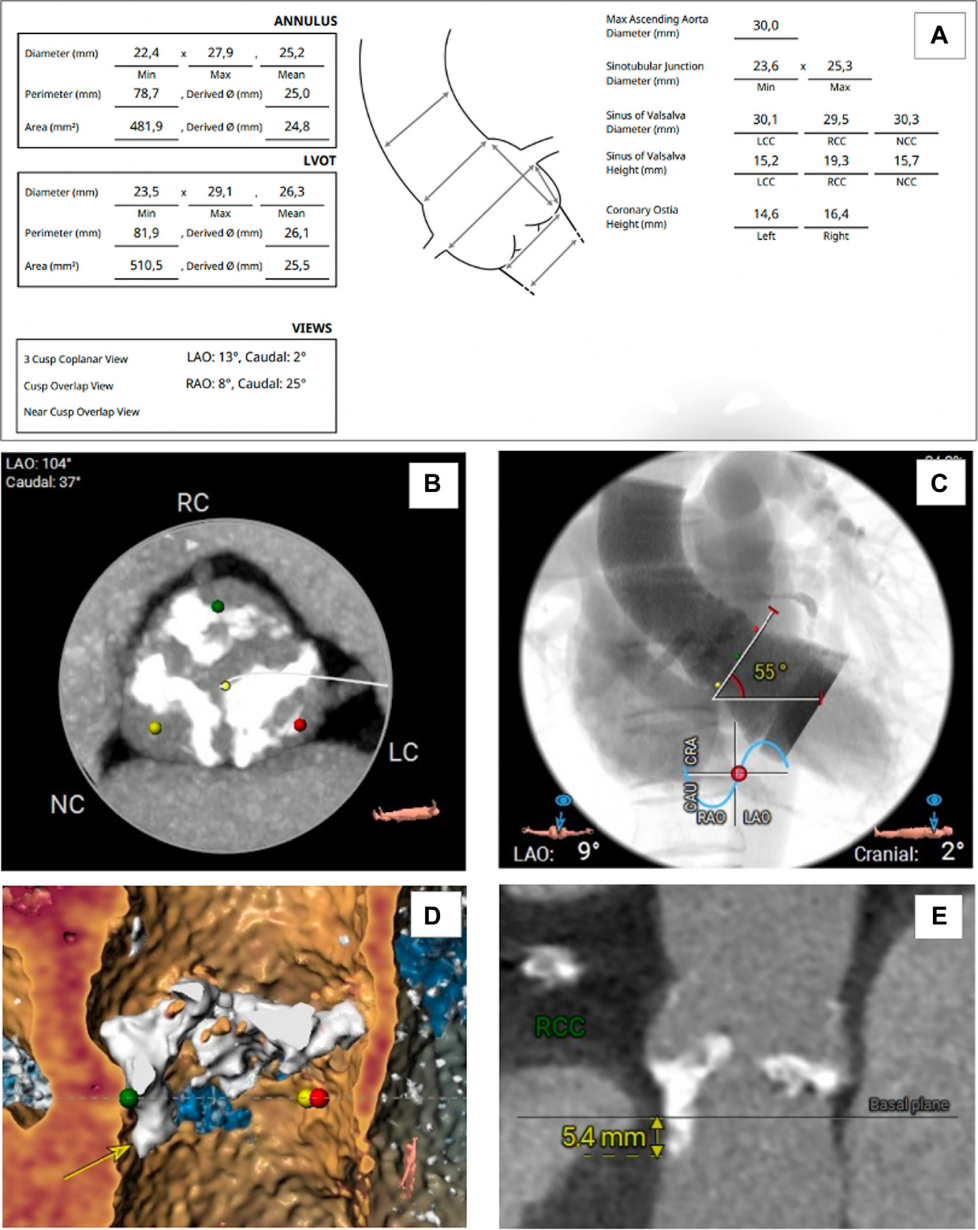
Cardiac and Aortic Computed Tomography (A) Measurements of the aortic annulus, left ventricular outflow tract, and aorta. (B) Heavily calcified aortic valve. (C) Aortic valve angulation relative to the horizontal plane. (D and E) Calcification in the left ventricular outflow tract (yellow arrow). CAU = caudal; CRA = cranial; LAO = left anterior oblique; LC = left coronary; LVOT = left ventricular outflow tract; Max = maximum; NC = noncoronary; RAO = right anterior oblique; RC = right coronary; RCC = right coronary cusp.

On the basis of the 2020 American College of Cardiology guidelines (Class 1, Level of Evidence: A), aortic valve replacement was indicated.^1^ Given the high surgical risk (The Society of Thoracic Surgeons score of 8.39% and European System for Cardiac Operative Risk Evaluation [EuroSCORE II] of 8.34%) and multiple comorbidities, the heart team recommended transcatheter aortic valve replacement (TAVR). Anatomical evaluations of the left ventricular outflow tract, aortic valve, and peripheral arteries (Figures 2A to 2E) supported the selection of a 29-mm Evolut R valve (Medtronic). The potential need for permanent pacemaker implantation was also considered given the preexisting left bundle branch block and significant calcification in the outflow tract.

## Management

The patient’s acute heart failure was stabilized using loop diuretic agents, nitroglycerin, and digoxin, resulting in symptom improvement, reduced dyspnea, and resolution of pulmonary congestion on chest radiography. On the seventh day of hospitalization, TAVR was performed using local anesthesia, with ultrasound-guided access through the bilateral femoral arteries and the left femoral vein. Coronary angiography confirmed no restenosis in the stents of the LAD artery and RCA (Video 1), and a temporary pacemaker was placed. Because of the high risk of recurrent cerebral hemorrhage, intravenous heparin was administered at a dose of 70 IU/kg.

A pigtail catheter was advanced into the Valsalva sinus to assess the aortic anatomy and valve function. Through a right femoral approach, an Amplatz Left 1 (AL-1) 6-F catheter was positioned at the aortic valve, and a 0.035-inch straight guidewire was carefully advanced across the valve. Following this, a pigtail catheter was used to measure left ventricular pressure and the transvalvular gradient, which showed a mean pressure gradient of 70 mm Hg. Subsequently, a 29-mm Evolut R valve was successfully deployed 2 to 3 mm below the virtual aortic annulus under fluoroscopic guidance.

**Figure 3:**
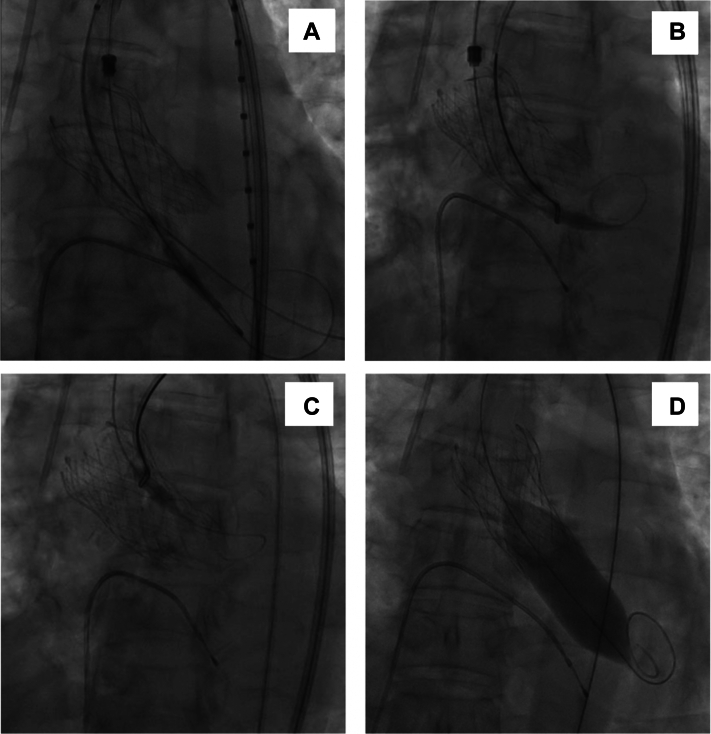
Diagnosis and Management of Nose Cone Entrapment (A) Infolding of the distal stent frame with entrapment of the nose cone. (B) Use of a snare to capture the nose cone. (C) Successful extraction of the nose cone from the delivery system. (D) Post-dilation performed using a 22-mm balloon.

During device retrieval, the Confida Brecker guidewire (Medtronic) became entangled at the proximal end of the stent frame, while the nose cone of the delivery catheter lodged at the distal end of the prosthesis (Figure 3A, Video 2). Additionally, infolding of the stent frame was observed at the site of a calcified nodule in the left ventricular outflow tract. Initial attempts to release the nose cone by using conventional maneuvers were unsuccessful. To address this issue, we used a Multi-Snare device (B. Braun) through the contralateral femoral artery access. This access site, established with a 7-F sheath, had been prepared earlier for placement of a 5-F pigtail catheter to facilitate aortic pressure monitoring. The snare device was delivered through a 7-F Judkins right (JR) 4.0 guiding catheter, which allowed precise navigation toward the entrapped nose cone. On successful engagement, gentle traction was applied, carefully dislodging the nose cone from the stent frame without damaging the prosthesis or adjacent structures (Figures 3B and 3C, Videos 3 and 4). Following its release, the nose cone was retracted into the delivery system, thus ensuring safe retrieval (Video 5). The snare and guiding catheter were then withdrawn, and the 5-F pigtail catheter was reintroduced to resume aortic pressure measurements. Subsequent angiography confirmed optimal valve positioning and the absence of device-related structural damage. Although a moderate paravalvular leak was identified, it was effectively addressed with post-dilatation using a 22-mm Z-MED II balloon (B. Braun) (Figure 3D). The procedure concluded with a significant reduction in the mean pressure gradient to 4 mm Hg.

Transthoracic echocardiography showed no evidence of pericardial effusion and only mild paravalvular regurgitation. Following the removal of all catheters and the temporary pacemaker, vascular closure was achieved with the Perclose ProGlide system (Abbott).

## Follow-Up

The patient showed marked clinical improvement following the procedure and was discharged 4 days later. At discharge, his medication regimen included dabigatran, rosuvastatin, and heart failure therapies: sacubitril/valsartan, metoprolol succinate, spironolactone, and dapagliflozin. At 3-month follow-up, the patient remained clinically stable, with a significant reduction in NT-proBNP to 170 ng/L. Transthoracic echocardiography confirmed proper positioning of the prosthetic valve and showed only mild paravalvular leakage and a mean pressure gradient of 3.29 mm Hg.

## Discussion

Nose cone entrapment during TAVR, although rare, is a serious complication with potential consequences such as paravalvular regurgitation, hemodynamic collapse, stroke, or cardiac arrest. Management of this complication is challenging because of limited data and relies heavily on operator experience. Forceful maneuvers to release the nose cone can worsen the situation by damaging the valve or surrounding structures.

A review of the literature revealed only 3 similar cases of nose cone entrapment, 2 managed by post-dilation and 1 managed by the snare technique.^2^, ^3^, ^4^ All cases involved significant aortic valve calcification, pronounced annular angulation, and a dilated aortic arch. In our case, multiple factors contributed to the entrapment, including severe calcification without pre-dilation and the presence of a large, calcified nodule in the left ventricular outflow tract that led to stent frame infolding. The patient’s 55° annular angulation and the use of a large, 29-mm valve further complicated the procedure.

In many cases, the snare-assisted technique is essential for positioning self-expanding valves in difficult anatomical settings by providing traction and centralization.^5^ However, in our case, the snare was used to free the nose cone entrapped between the prosthetic valve and the calcified left ventricular outflow tract. Post-dilation would have required a stiff wire and large-bore access, with unpredictable outcomes, whereas the snare could be deployed through the existing catheter system, thereby minimizing access-related complications and improving control. This approach ensured prompt resolution and reduced procedural risks.

To prevent nose cone entrapment, recognizing and addressing risk factors are critical to improving procedural safety. These risk factors include severe aortic valve calcification, excessive annular angulation, valve oversizing, absence of pre-dilatation, and suboptimal device manipulation during deployment or retrieval. Extra caution is needed when using large-diameter, second-generation self-expanding valves (29 mm and 34 mm) because they are more prone to complications such as stent frame infolding.^6^ Preventive strategies should include considering pre-dilatation in patients with heavily calcified valves or prominent calcified nodules in the left ventricular outflow tract, using meticulous valve sizing tailored to the patient’s anatomy, and performing careful fluoroscopic assessment during device retrieval to detect early signs of stent frame infolding. Ensuring that these procedures are conducted by experienced operators is essential, particularly in patients with high-risk anatomies. In cases of entrapment, the snare technique provides a reliable and effective solution for device retrieval.

## Conclusions

This case highlights the successful use of the snare technique in resolving nose cone entrapment during TAVR, offering precise control for a safe and efficient solution to this rare complication.

## Funding Support and Author Disclosures

The authors have reported that they have no relationships relevant to the contents of this paper to disclose.

## Appendix

Video 1 Coronary Angiography

Video 2 Infolding of the Distal Stent Frame With Nose Cone Entrapment

Video 3 Snare Capture of the Entrapped Nose Cone

Video 4 Extraction of the Nose Cone From The Prosthetic Valve

Video 5 Retrieval of the Nose Cone Into the Delivery System
